# Whole genome structure and resistance genes in carbapenemase-producing multidrug resistant ST378 *Klebsiella pneumoniae*

**DOI:** 10.1186/s12866-023-03074-7

**Published:** 2023-11-03

**Authors:** Su Min Kyung, Jun Ho Lee, Eun-Seo Lee, Cheol-Yong Hwang, Han Sang Yoo

**Affiliations:** 1 Department of Infectious Disease, College of Veterinary Medicine, Seoul National University, Seoul, Republic of Korea; 2 Department of Veterinary Dermatology, College of Veterinary Medicine, Seoul National University, Seoul, Republic of Korea

**Keywords:** Enterobacterales, Carbapenemase, Metallo-β-Lactamase, NDM-5, *Klebsiella pneumoniae*, Epidemiology

## Abstract

**Background:**

Carbapenemase-producing *Klebsiella pneumoniae* (CPKP) is one of the most dangerous multidrug-resistant (MDR) pathogens in human health due to its widespread circulation in the nosocomial environment. CPKP carried by companion dogs, which are close to human beings, should be considered a common threat to public health. However, CPKP dissemination through companion animals is still under consideration of major diagnosis and surveillance systems.

**Methods:**

Two CPKP isolates which were genotyped to harbor *bla*_NDM-5_-encoding IncX3 plasmids, were subjected to the whole-genome study. Whole bacterial DNA was isolated, sequenced, and assembled with Oxford Nanopore long reads and corrected with short reads from the Illumina NovaSeq 6000 platform. The whole-genome structure and positions of antimicrobial resistance (AMR) genes were identified and visualized using CGView. Worldwide datasets were downloaded from the NCBI GenBank database for whole-genome comparative analysis. The whole-genome phylogenetic analysis was constructed using the identified whole-chromosome SNP sites from *K. pneumoniae* HS11286.

**Results:**

As a result of the whole-genome identification, 4 heterogenous plasmids and a single chromosome were identified, each carrying various AMR genes. Multiple novel structures were identified from the AMR genes, coupled with mobile gene elements (MGE). The comparative whole-genome epidemiology revealed that ST378 *K. pneumoniae* is a novel type of CPKP, carrying a higher prevalence of AMR genes.

**Conclusions:**

The characterized whole-genome analysis of this study shows the emergence of a novel type of CPKP strain carrying various AMR genes with variated genomic structures. The presented data in this study show the necessity to develop additional surveillance programs and control measures for a novel type of CPKP strain.

**Supplementary Information:**

The online version contains supplementary material available at 10.1186/s12866-023-03074-7.

## Background

*Klebsiella pneumoniae* is one of the most notable pathogens worldwide, causing various infections such as bloodstream infections, urinary tract infections and liver abscesses [1]. Neonatal, elderly and immunocompromised nosocomial patients are particularly vulnerable to these infections [2], posing a threat to public health in our society. Carbapenem is considered the last resort antibiotic prescribed for infections by *K. pneumoniae*, but the emergence of carbapenemase-producing *K. pneumoniae* (CPKP) greatly limits this treatment option in medical situations. Carbapenemase production induces resistance not only against carbapenems but also to more than 2–3 classes of antibiotics [3], leading to dramatically reduced treatment options and increased mortality, ranging from 18.9% to 48.0% for carbapenem-resistant Enterobacterales (CRE) infections [4–6]. As a consequence of the overuse of carbapenems, carbapenemase-producing Enterobacterales (CPE) strains have been disseminated worldwide in modern society [7].

Enterobacterales strains can acquire resistance against carbapenems via three major mechanisms: the production of enzymes such as carbapenemase, porin loss and overexpression of efflux pumps [8, 9]. Although carbapenemase production is the most powerful mechanism, two other pathways can also provide resistance against carbapenems, either solely or in combination with carbapenemase production [10, 11]. A major concern is the coexistence of multiple resistance mechanisms in a single strain, which could result in synergism and lead to even higher levels of resistance.

A nationwide study conducted in our country [12] showed that the circulation of CPE in the human nosocomial environment was mostly associated with *K. pneumoniae* ST11 and ST307. In contrast to the results from human investigations, the first detected animal-derived CPKP in our country was identified as ST378 *K. pneumoniae* [13]. ST378 *K. pneumoniae* has never been reported as a CPKP anywhere. In the genomic characterization of ST378 *K. pneumoniae* conducted in the previous study [13], the identified information was limited to the *bla*_NDM-5_ gene-encoded IncX3 plasmid. However, the isolate was found to have resistance against various classes of antimicrobial agents. Therefore, further genomic analysis was necessary to characterize the ST378 isolates.

In this study, genomic characterization was conducted for the isolates using a whole-genome approach. The genomic analysis was conducted to reveal antimicrobial resistance (AMR) genes from the whole-genome and to identify epidemiologic information for the isolates.

## Methods

### The whole-genome sequencing, de novo assembly and annotation

Two strains of ST378 *K. pneumoniae* (DMCPKP1 and DMCPKP4), confirmed to contain the *bla*_NDM-5_ gene-encoded IncX3 plasmid, were used for the analysis [13]. The bacterial isolates were originated from urine samples of Korean dogs hospitalized in Seoul National University. For genome sequencing, DNA purification was performed using a Wizard Genomic DNA Purification Kit (Promega, Madison, WI) from overnight cultures. Two independent genomic DNA libraries were prepared for both short and long read systems and sequenced using Illumina NovaSeq 6000 (Illumina, San Diego, CA, USA) following a paired-end 2 × 150-bp protocol and Oxford Nanopore (Oxford Nanopore Technologies, Oxford, UK) platforms. Guppy basecaller and barcoder v6.0.7 were used for basecalling and demultiplexing all sequencing runs. Reads were trimmed and filtered for long and high-quality reads using FiltLong v0.2.0 to match the raw data to the appropriate data size for assembly. Filtering of read data to proceed with de novo assembly was conducted using Flye v2.8.3 [14]. Assembly of both Illumina and Oxford Nanopore long reads was performed using the Unicycler v0.4.8 hybrid assembler [15]. Circlator v1.5.5 was used to determine whether the assembly resulted in a circular form or linear form [16]. Polishing work was carried out with Pilon v1.23 [17] for the contigs whose structural shape was identified, and the evaluation of the assembly results was done using BUSCO v4.1.2 [18]. After polishing, structural annotation was performed using Prokka v1.14.6 [19] and functional annotation was performed with DIAMOND v 0.9.30 [20]. Gene Ontology (GO) [21] annotation was performed based on Blast2GO v4.1.9 [22].

### Identification and visualization of characteristic genes from the whole-genome datasets

Antibiotic resistance genes, virulence genes and plasmid replicons were identified using the ResFinder [23] and PlasmidFinder [24] databases available on the Center for Genomic Epidemiology (CGE) server (http://www.genomicepidemiology.org). Mobile gene elements (MGEs) and point mutations were also investigated with MobileElementFinder [25] and PointFinder [26]. The presence of spacers of CRISPR-Cas systems was identified using CRISPRCasFinder 4.2.20 with default parameters [27]. All detected and identified antimicrobial resistance genes were compared to sequences available from the National Center for Biotechnology Information. The schematic mapping was visualized using CGView [28].

### Bioinformatic analysis of the available whole-genome datasets

For comparative whole genome analysis, 67 *K. pneumoniae* whole genome datasets described in previous articles were accessed from NCBI GenBank database. Since there were no available ST378 *K. pneumoniae* whole-genome datasets to compare, worldwide *K. pneumoniae* datasets were used as reference datasets. The high quality SNP calling, filtering, and site validation were conducted using a web-based tool with default parameters [29], based on *K. pneumoniae* HS11286 (Genbank accession no. CP003200.1) as the reference. Maximum likelihood (ML) phylogenetic tree was constructed with 1000 bootstrap values using MEGA-11 software [30]. An antimicrobial resistance gene distribution heatmap was generated using the interactive tree of life (iTOLs) tool. Epidemiological profile was denoted and displayed alongside the phylogenetic tree on the same server. In silico multi-locus sequence typing (MLST) using seven housekeeping genes (*adk*, *fumC*, *gyrB*, *icd*, *mdh*, *purA* and *recA*) was conducted for the strains in this study and all chromosomal datasets downloaded from GenBank, using MLST 2.0 [31] available on the CGE server.

## Results

### General profiles of the sequenced and assembled whole-genomes of CPKP isolates

As a result of sequencing, high-quality assembled contig records longer than 5,290,663 base pairs were generated, and the total GC content of both strains was about 57%. As a result of the alignment, over 97% of the base reads were successfully mapped. The quality of sequencing and mapped reads was presented in Supplementary Table S1. Each strain was found to carry a single chromosome, and four plasmids were identified from both strains (Table 1). Multiple plasmids were identified from both isolates, featuring various lengths ranging from 46 to 456 kbp.

**Table 1:** Identified genome profiles from *K. pneumoniae* isolates

Bacterial Strain	Gene type (name)	Gene Length (bp)	GC contents	CDS	rRNA	tRNA
**DMCPKP1**	Chromosome	5,291,572	57.45	4,886	25	89
	Plasmid (pKP1_IncHI1B/IncFIB)	456,420	48.04	529	0	0
	Plasmid (pKP1gtc3L95)	95,047	52.4	111	0	0
	Plasmid (pKP1gtc5L70)	70,930	51.05	84	0	0
	Plasmid (pKP1-NDM5)	46,112	47.04	58	0	0
**DMCPKP4**	Chromosome	5,290,663	57.45	4,896	25	89
	Plasmid (pKP4_IncHI1B/IncFIB)	434,340	48.04	468	0	0
	Plasmid (pKP4gtc3L95)	95,046	52.4	107	0	0
	Plasmid (pKP4gtc4L70)	70,932	51.05	84	0	0
	Plasmid (pKP4-NDM5)	46,112	47.04	58	0	0

As a result of the sequencing and assembly, multiple gene components were identified. Each strain was identified to contain single chromosome and 4 plasmids, respectively. Six different types were discovered from four heterogenous plasmids. Three types (IncFIB(pNDM-Mar), IncHI1B(pNDM-MAR) and IncFIB(K)) of plasmids were discovered as integrated form in the pKP1_IncHI1B/IncFIB and pKP4_IncHI1B/IncFIB

The corresponding minimum inhibitory concentration (MIC) phenotype profiles obtained in the previous study [13] are presented in Supplementary Table S2 .

### Characterization of the chromosomes of the CPKP isolates

**Fig. 1:**
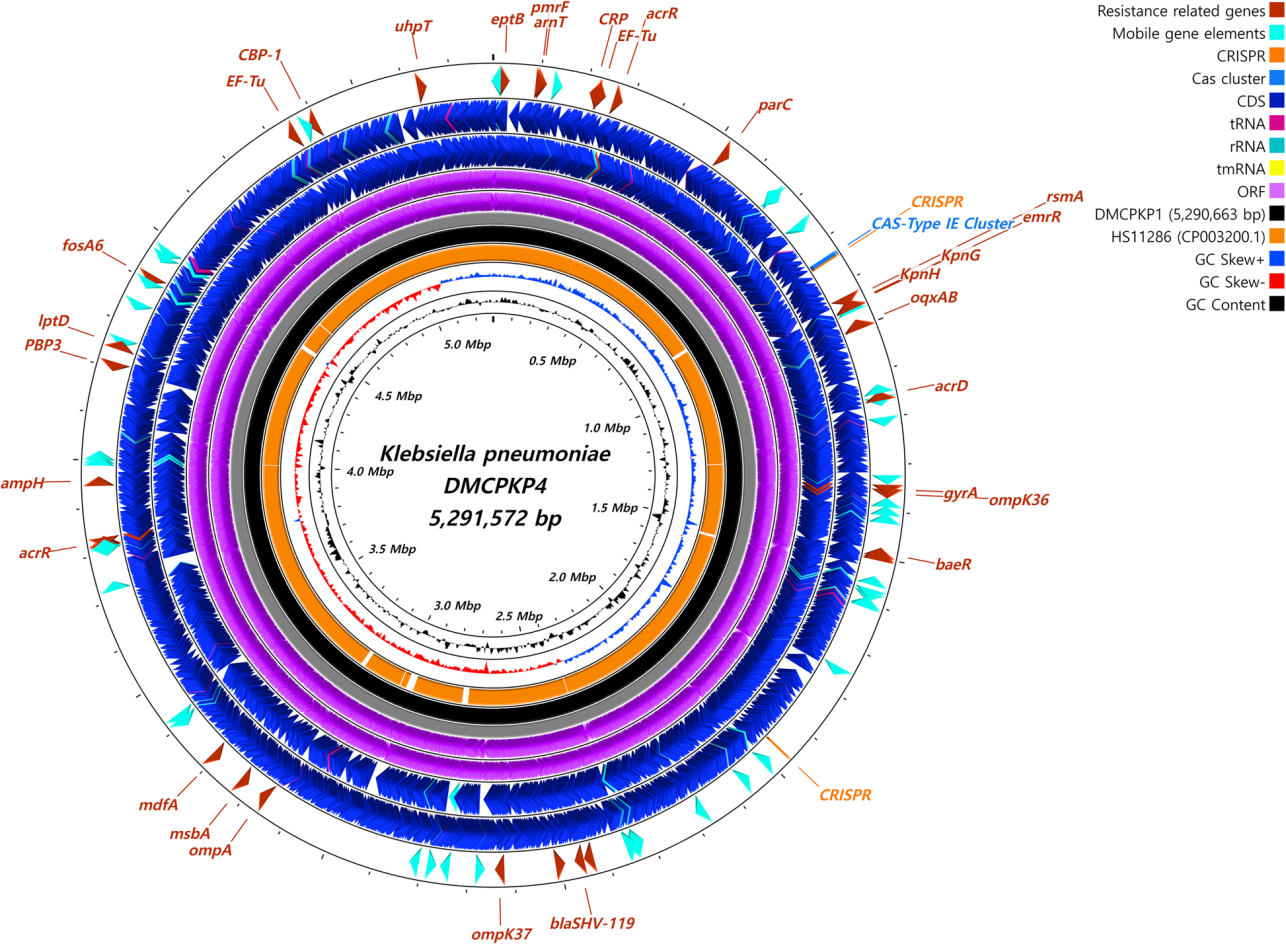
Schematic circular map of whole-chromosomes identified from CPKP isolates compared with HS11286. The whole chromosomes of DMCPKP1 (5,290,663 base pair long) and DMCPKP4 (5,291,572 base pair long) were identified in the study and visualized. Characteristic genes, including resistance genes, mobile genes and CDS sites were marked with distinguishable colors. The whole-genome data of *K. pneumoniae* HS11286 (GenBank accession no. CP003200.1) was used as a reference and represented as a black circle. The circular map was visualized using CGView

The chromosome of each CPKP strain consisted of 5.29 MB of genes (Fig. 1). DMCPKP4 had a slightly longer chromosome than DMCPKP1. The chromosomes of DMCPKP1 and DMCPKP4 contained 4,886 and 4,896 CDS encoding sites, respectively. Both isolates were identified to carry *bla*_SHV-119_ in their chromosomes. The chromosomes were also found to have various mutated sites that contribute to AMR, including *ompK36*, *ompK37*, *acrR*, *gyrA* and *parC*. Genomic sites of *ompK35, gyrB,* and *rpsL* were found without mutations.

The *ompK36* gene was found to have multiple amino acid substitutions (3 sites of deletion and 17 sites of substitution) from the *ompK36* gene of *K. pneumoniae* isolate 1,537,514 (accessed as GenBank no. KY086540.1). The complete CDS of this gene was matched with 100% identity and query coverage via a BLAST search with the *opmK36* gene of *K. pneumoniae* strain 487,881 (GenBank no. MG576697.1). Moreover, *ompK37* was found to have 2-point mutations (G174_TAA-CAA, T330_GAC-AAC) from the reference gene accessed as GenBank no. KC534862.1, but there were no amino acid sequence variations.

**Fig. 2:**
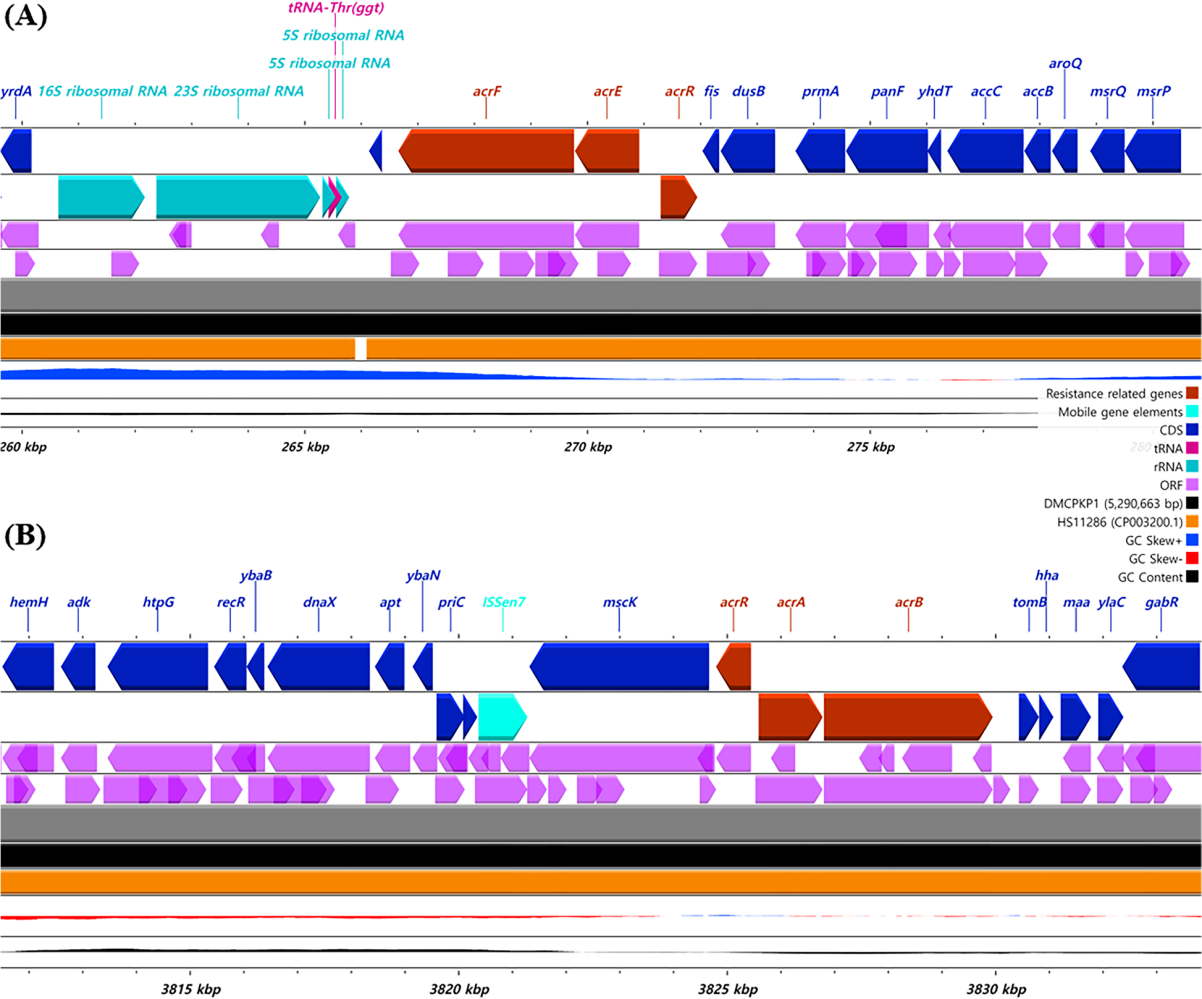
The identified gene structure region carrying the mutated *acrR* on the chromosome of the CPKP isolates. The mutated structure around the acrR gene was identified in the study and visualized. The gene region around *acrR* was found to be identical in both strains of CPKP. The transcriptional regulator operon acrR was found to be closely related to either the *acrRAB* or *acrREF* structure. The regional genetic structure was identical for both strains. *K. pneumoniae* HS11286 was comparatively marked as a horizontal map. The gene structure was visualized using CGView

The gene *acrR* in the transcriptional repressor operon of the multidrug efflux transporter was found to be closely linked with either *acrEF* (Fig. 2A) or *acrAB* (Fig. 2B). The *acrR* gene linked with *acrAB* was found to have 31 base pairs mutated at 3 regions in the amino acid sequence (F115Y, I116V and P216S) from previously accessed *K. pneumoniae acrR* (GenBank no. CP065838.1). The *acrR* gene linked with *acrEF* was identical to the previously accessed acrEF/envCD operon transcriptional regulator product domain (GenBank no. CP034540.1). The gene structure areas around these operons are presented in Fig. 2.

**Fig. 3:**
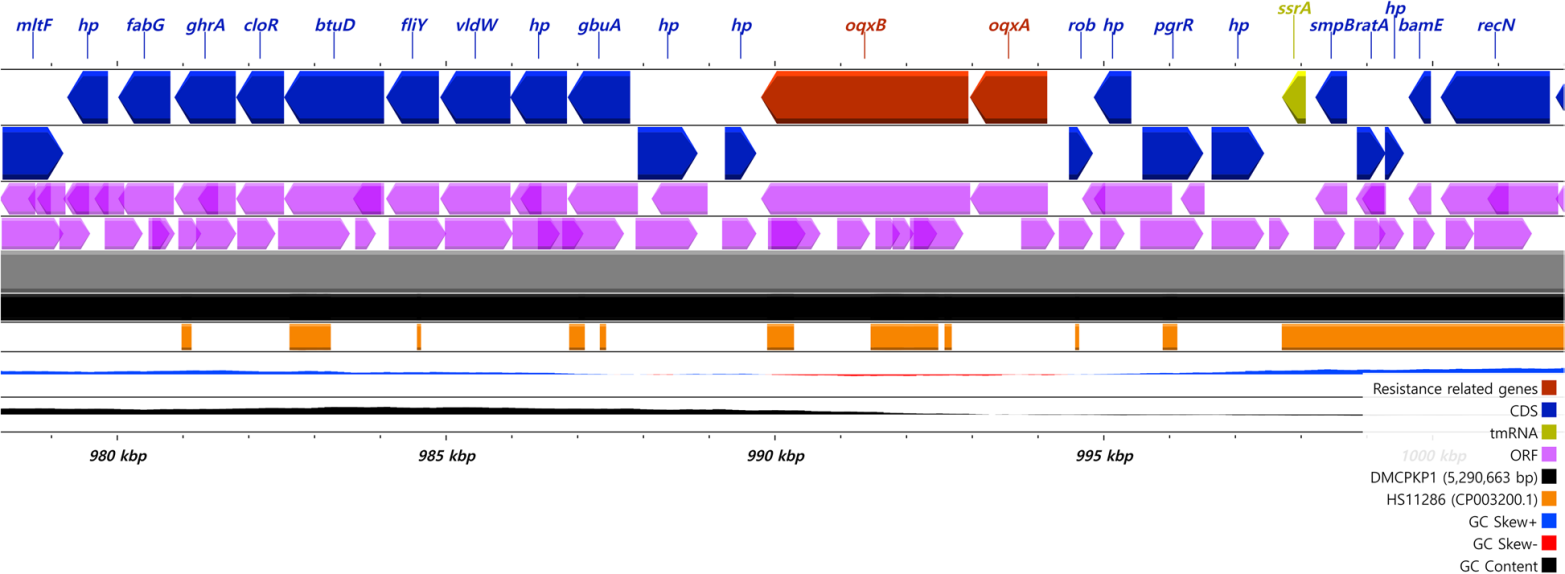
Linear structure visualization around the mutated efflux pump expression gene *oqxAB*. The gene structure was revealed to be mutated and visualized using CGView. DMCPKP1 and DMCPKP4 had the identical *oqxAB* gene structures. Gene sequence from *K. pneumoniae* HS11286 was depicted in comparison in a horizontal comparison

The multidrug efflux RND transporter periplasmic adaptor subunit *oqxA* and permease subunit region *oqxB* were also discovered on the chromosome, which is known as *oqxAB* in a combined form (Fig. 3). The regional gene structure around *oqxAB* was identical in both strains, as mapped in Fig. 3. Additionally, novel mutation sites were discovered in both the *oqxA* and *oqxB* genes that were not previously identified. The multidrug efflux RND transporter periplasmic adaptor subunit *oqxA* was found to have 2 base pair mutations (comparative gene GenBank accession no. CP098169.1) but no amino acid sequence substitutions. Moreover, the multidrug efflux RND transporter permease subunit *oqxB* was found with 12 mutation sites and a substitution of M881V compared to the NCBI reference gene *oqxB26*.

The DNA gyrase subunit *gyrA* was identified in the chromosome, with 82-point mutation sites and 4 amino acid sequence variations (comparative gene GenBank no. CP065838.1). This type of mutation has never been reported or identified before. A previously unreported variation was also identified from the DNA topoisomerase IV subunit *parC*, with a mutated amino acid site of S81I. These types of mutations can confer additional resistance against antimicrobial classes of fluoroquinolone.

A new variation in the resistance gene *fosA*, which can confer resistance against Fosfomycin, was also discovered on the chromosome, with 96.7% identity compared to published data. The genetic sequence of mutated *fosA* in both strains was identical.

### Identified plasmids from the CPKP isolates

**Fig. 4:**
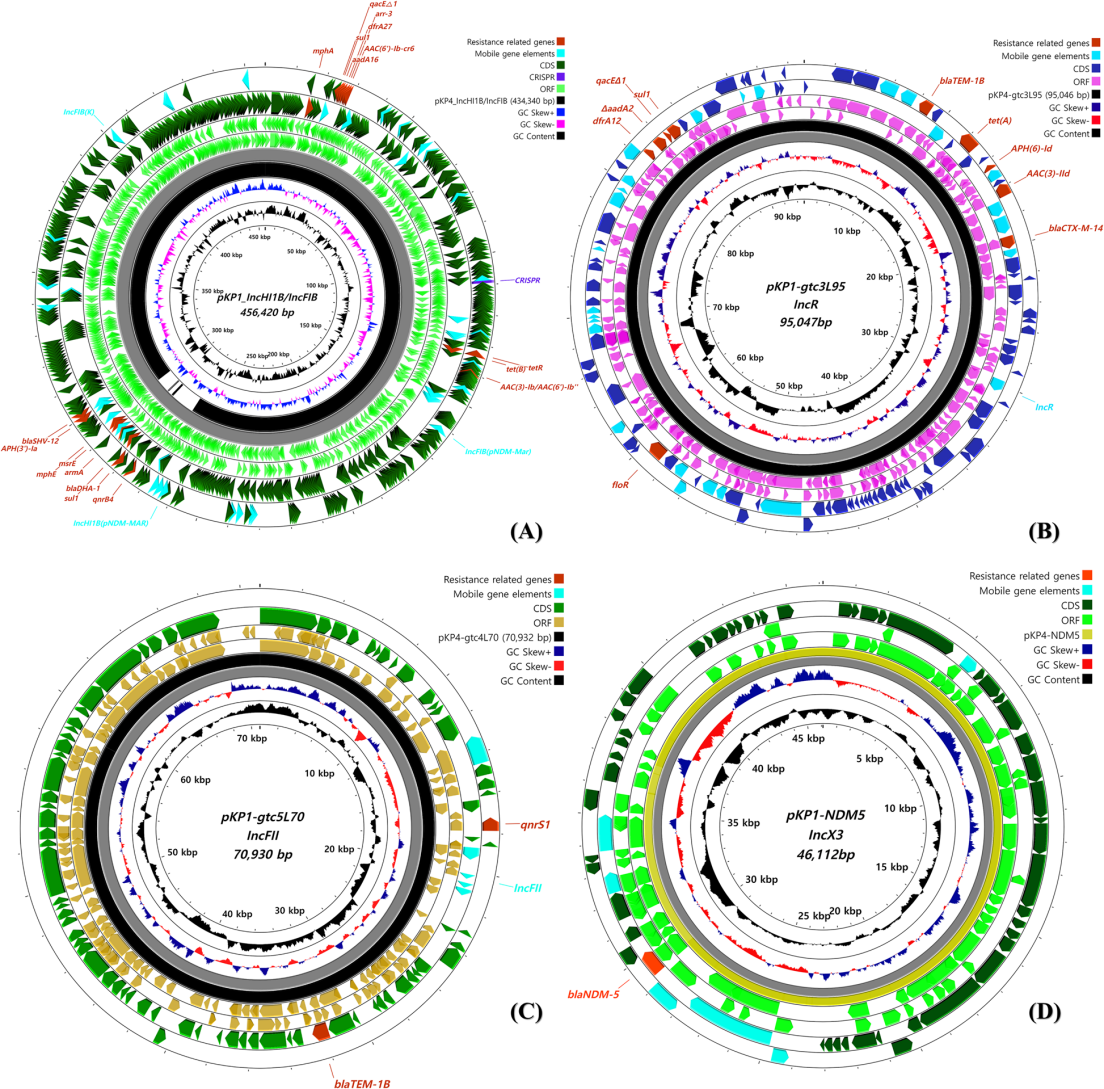
Comparative visualization of the identified plasmids from the CPKP isolates. Four heterogenous plasmids were assembled from the isolates, each carrying resistance genes. **A** A novel plasmid structure with three integrated types of plasmids, namely IncFIB(pNDM-Mar), IncHI1B(pNDM-MAR), and IncFIB(K), was identified. Furthermore, additional plasmid types of (**B**) IncR, (**C**) IncFII and (**D**) IncX3 were found identical from both CPKP strains. Each plasmid was identified to carry β-lactamase genes, either harboring ESBLs (*bla*_*SHV-12*_, *bla*_*CTX-M-14*_ and *bla*_*TEM-1B*_) or carbapenemase (*bla*_*NDM-5*_)

Multiple plasmids encoding various antimicrobial resistance genes were identified from both CPKP strains. Four plasmids were assembled from both strains, ranging from 46–465 kbp (Fig. 4A-D). Plasmid types of IncHI1B (pNDM-MAR), IncFIB (K), IncFIB (pNDM-Mar), IncR, IncFII and IncX3 were identified from both strains. All plasmids were found to contain antibiotic resistance β-lactamase genes, including the carbapenemase *bla*_NDM-5_ carried by the IncX3 plasmid. Two sites of ESBL encoding *bla*_TEM-1B_ gene were discovered from two different plasmids, IncR and IncFII.

The plasmid pKP1-IncHIB/IncFIB (Fig. 4A) was sequenced and assembled with 529 CDS encoding sites and 48.04% GC content (Table 1). Three plasmid types were identified in the pKP1-IncHIB/IncFIB, namely IncFIB(pNDM-Mar), IncHI1B(pNDM-MAR) and IncFIB(K). The pKP4-IncHIB/IncFIB, identified from DMCPKP4 was assembled 22,080 base-pair shorter than pKP1-IncHIB/IncFIB. Various AMR genes were identified from this plasmid, which would confer multidrug resistance (MDR) with the plasmid alone. The contributable antimicrobial agents from this plasmid included: aminoglycosides (*aadA16*, *aac(6’)lb-cr*, *armA* and *aph(3’)-la*), tetracyclines (*tet(B)*), macrolides (*mphA*, *mphE* and *msrE*), quinolones (*aac(6’)lb-cr* and *qnrB4*), folate pathway antagonists (*sul1* and *dfrA27*), rifamycins (*arr-3*), aminocyclitols (*aadA16*) and β-lactams (*bla*_*DHA-1*_ and *bla*_*SHV-12*_). The plasmid carried by DMCPKP1 (pKP1-IncHIB/IncFIB) was found with additional resistance genes of *qnrB4, bla*_*DHA-1*_*, armA, msrE* and *mphE*.

The IncR type plasmid pKP1-gtc3L95 was sequenced and was as long as 95,047 bp with 111 CDS encoding sites and 52.4% of GC content, whereas pKP4-gtc3L95 was a single base pair shorter with 107 CDS encoding sites (Fig. 4B). The plasmid encoded multiple resistance genes, including extended-spectrum β-lactamase (ESBL) which could be enough to confer MDR capability to the organism. The AMR genes carried by the IncR plasmid were able to contribute to resistance against various classes of drugs, such as aminoglycosides (*aph(6)-Id* and *aac(3)-IId*), phenicols (*floR*), sulphonamides (*sul1*), tetracyclines (*tet(A)* and *tetR*), trimethoprims (*dfrA12*) and β-lactams (*bla*_*CTX-M-14*_ and *bla*_*TEM-1B*_). Notably, a gene structure carrying multiple AMR genes coupled with MGEs was identified; IS*1663-bla*_*TEM-1B*_*-tnpR-*Tn*2-tet(A)-tetR-aph(6)Id-*IS*26-aac(3)IId-*IS*Vsa5-*IS*Vsa5- bla*_*CTX-M-14*_*-*IS*903*.

pKP1-gtc5L70 was sequenced and identified as a 70,930 base pair long plasmid (Fig. 4C), featuring 51.05% GC content and containing 84 CDS encoding sites. An identical plasmid, pKP4-gtc4L70, was identified from DMCPKP4, featuring sequences as long as 70,932 base pairs with 84 CDS encoding sites. Both plasmids were typed as incompatibility type IncFII. The IncFII plasmids contained broad-spectrum class A β-lactamase encoding gene *bla*_TEM-1B_ and quinolone resistance pentapeptide repeat protein *qnrS1*. The ESBL-encoding gene *bla*_TEM-1B_ was found with a variation, with 99.77% identity from that of the reference accession gene no. AY458016.

The IncX3 plasmids pKP1-NDM5 and pKP4-NDM5, which were characterized in the previous study [13], were also reidentified as a result of the sequencing and assembly (Fig. 4D). The plasmids were identified as long as 46,112 base pairs long with 122 CDS encoding regions, carrying the subclass B1 metallo-β-lactamase NDM-5 encoding *bla*_NDM-5_ gene.

The genome sequences of the chromosome and plasmids of DMCPKP1 and DMCPKP4 have been deposited in NCBI GenBank under BioProject accession number PRJNA858561.

### Bioinformatic resistance gene distribution comparison with whole genome phylogeny

Epidemiological analysis was conducted using previously described *K*. *pneumoniae* whole-genome datasets accessible from GenBank (Supplementary Table S3). A total of 67 strains of accessible whole-genome datasets from GenBank, including *K. pneumoniae* HS11286 (GenBank no. CP003200.1), were identified and compared with 2 datasets from this study. As a result of characteristic gene identification, a total of 114 AMR genes (Supplementary Table S4) and 27 types of plasmids (Supplementary Table S5) were discovered from the *K. pneumoniae* datasets. Among resistance genes, the aminoglycoside acetyltransferase *aac(6')-Ib-cr* gene was carried by 28 strains in this study, which is known for conferring ciprofloxacin resistance [32]. The aminoglycoside phosphotransferase *aph(3'')-Ib* was carried by 20 strains of *K. pneumoniae*, and 50% of the genes were identified as a modified form. Among β-lactamases, *bla*_*TEM-1B*_ and *bla*_*SHV-182*_ were found in 24 and 21 *K. pneumoniae* strains, respectively. The *bla*_*KPC-2*_ and *bla*_*NDM-1*_, known as carbapenemase genes, were also identified in 18 and 13 strains, respectively. The *bla*_*NDM-5*_-harboring ST378 *K. pneumoniae* strains carried *aac(6')-Ib-cr* and *bla*_*TEM-1B*_, but did not carry *aph(3'')-Ib*, *bla*_*SHV-182*_, *bla*_*KPC-2*_ or *bla*_*NDM-1*_. Plasmids were identified from 50 whole-genome datasets from GenBank. Among the plasmids, IncFIB(K) and IncR were the most frequently discovered types. These plasmids were also found in ST378 *K. pneumoniae* strains.

**Fig. 5:**
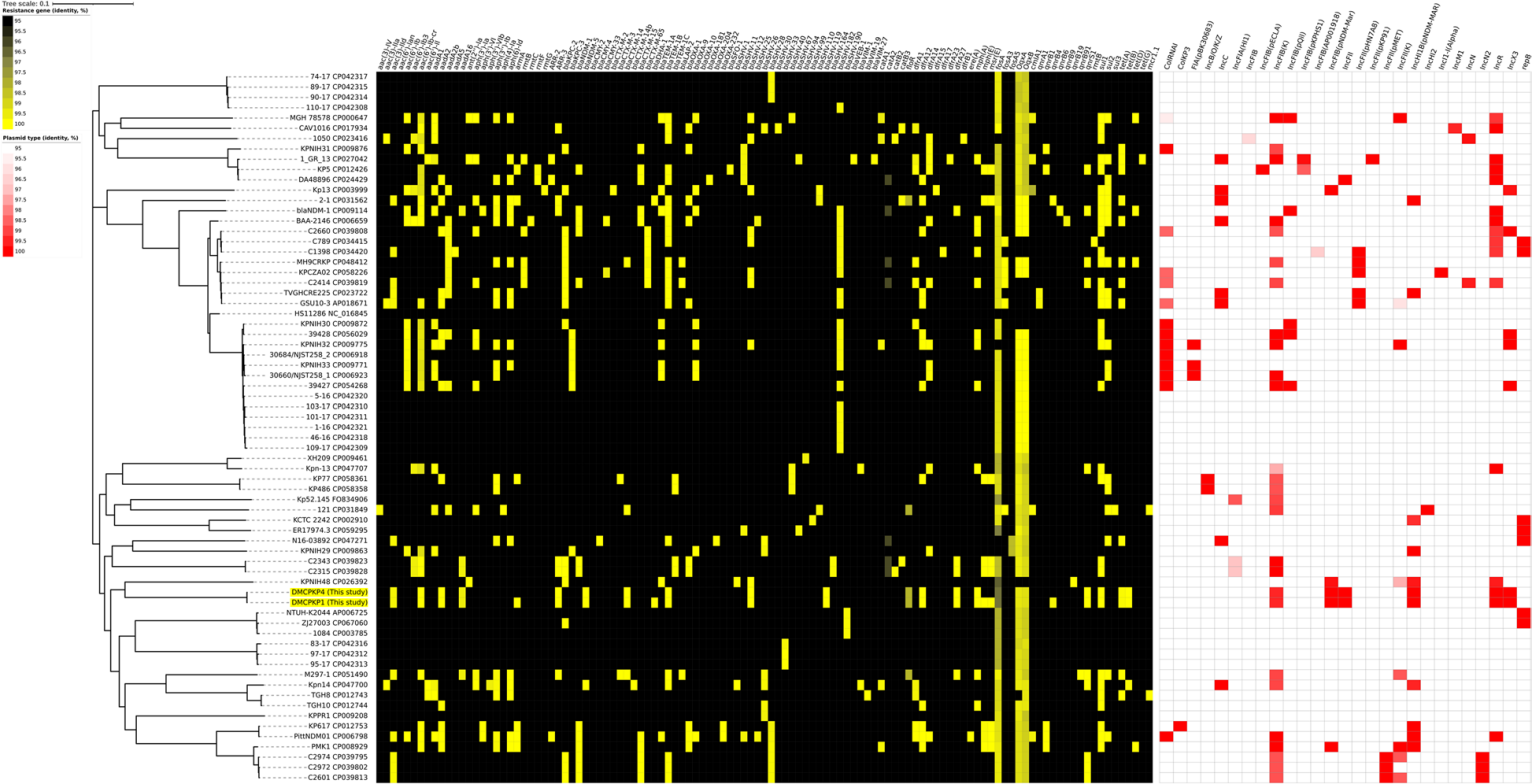
Phylogenetic analysis with AMR genes and plasmid types of *K. pneumoniae* whole-genome datasets. The whole-genome phylogenetic tree comparing 69 strains was constructed based on whole-genome SNPs from *K. pneumoniae* HS11286. The ST378 *K. pneumoniae* isolates of this study were highlighted with yellow background. The AMR gene prevalence was presented as yellow heatmap. The plasmid incompatibility types were presented as read heatmap. The resistance gene and plasmid type metadata listed in this heatmap are presented in Supplementary Table S4 and Supplementary Table S5. The phylogenetic tree was visualized using the iTOLs

The identified pairwise whole-chromosome SNP difference (Supplementary Table S6) between the datasets ranged from 0 to 17,719. The smallest SNP difference (0 point) was found between strain DMCPKP1 and DMCPKP4 from this study, followed by a 2-point difference identified between strains C2601 (GenBank no. CP039813.1) and C2972 (GenBank no. CP039802.1). The ST378 strains were identified to have SNP differences ranging from 15,651 to 17,139 compared to other strains, indicating their phylogenetic distance from other *K. pneumoniae* datasets. The ML phylogenetic tree was constructed using the whole-chromosome SNPs, and visualized with the characterized AMR genes and plasmid types in Fig. 5.

**Fig. 6:**
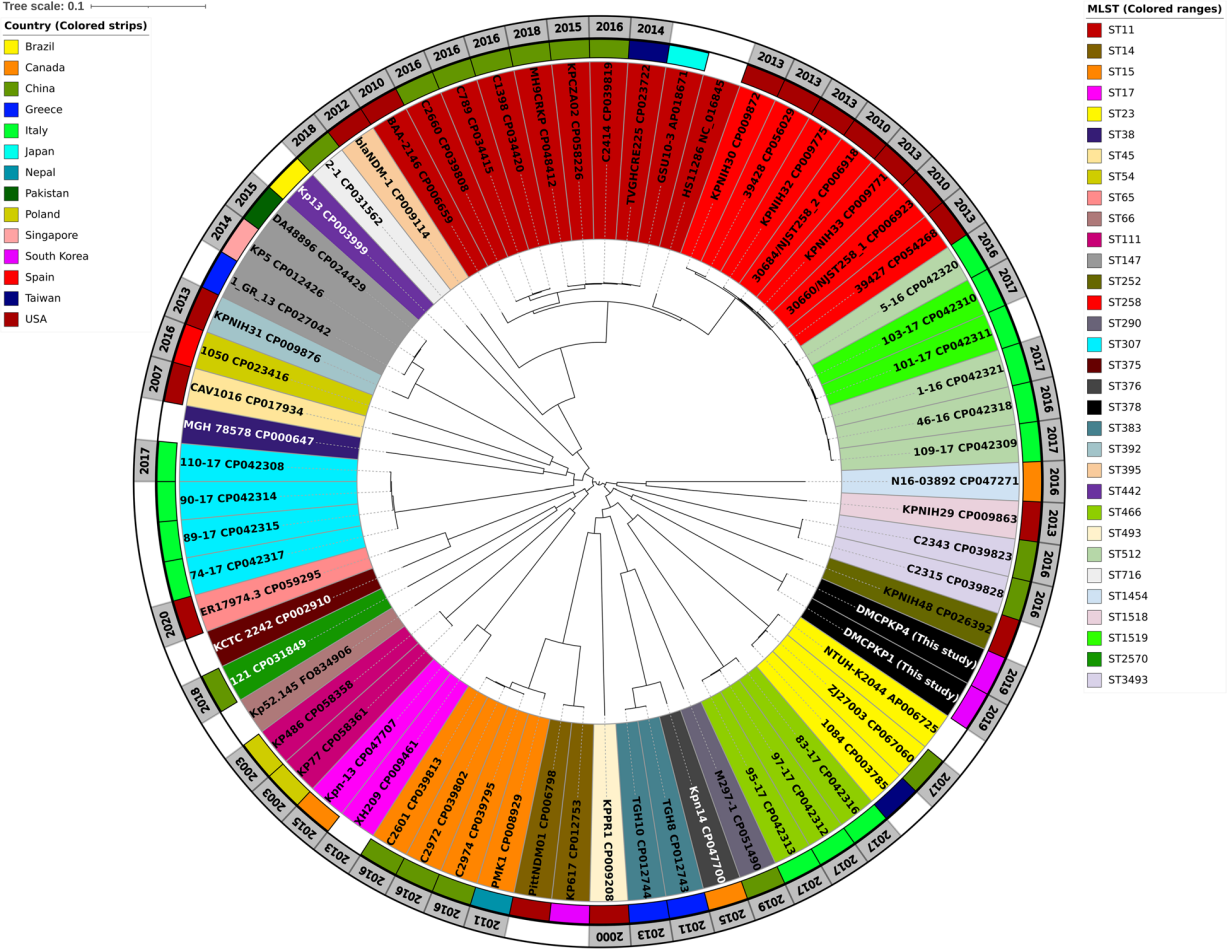
Epidemiologic profiles with whole-genome phylogeny of *K. pneumoniae* strains. The epidemiological data describing MLST types, isolated countries and isolative years were depicted with whole-genome phylogenetic tree of *K. pneumoniae* strains. The colored ranges around the strain label indicate MLST types. The inner colored strips indicate the isolated country of the strains. The grey colored outer strips indicate the isolated year of the strains. The epidemiological map was visualized using the iTOLs

Epidemiological profiles of 69 *K. pneumoniae* strains were presented along with the phylogenetic tree (Fig. 6). Isolated country, isolation year and MLST data are displayed with distinguishable colors and strips. The identified *K. pneumoniae* strains from 14 different countries and 32 different MLST types were visualized. A total of 16 whole-genome datasets from the USA and China were included in the study, which were the countries with the highest inclusion, followed by 13 datasets reported from Italy. The most frequently included MLST types in the analysis were ST11 and ST258. The whole-genome phylogenetic tree displayed the distance of the ST378 strains from other datasets, confirming that the isolates constitute a novel type of CPKP.

## Discussion

The AMR genes identified through whole-genome analysis of ST378 *K. pneumoniae* are well known for contributing to MDR capability against various classes of antimicrobial agents. High resistance levels against various classes of antimicrobial agents were confirmed (Supplementary Table S2) in the MIC level measurements conducted in a previous study [13]. One of the aims of the whole-genome analysis conducted this study was to identify the genomic origin contributing to the broad spectrum of AMR capability of ST378 isolates. In the whole-genome analysis, AMR genes contributable against various classes of antimicrobial agents were identified, namely aminoglycosides (*armA, aac(6’)-lb-cr, aac(3)-lld*), aminocyclitols (*aadA16* and *aadA2*), β-lactams (*bla*_NDM-5_, *bla*_SHV-12_, *bla*_SHV-119_, *bla*_DHA-1_, *bla*_TEM-1B_ and *bla*_CTX-M-14_), folate pathway antagonists (*sul1, oqxA, oqxB, dfrA12, dfrA27*), fosfomycins (*fosA*), macrolides (*mph(A)* and *msr(E)*), quinolones (*aac(6’)-lb-cr, acrR, qnrB4, qnrS1, gyrA* and *parC*), rifamycin (*ARR-3*), streptogramin B (*msr(E)*) and tetracyclines (*tet(A)* and *tet(B)*). The confirmed MIC levels of ST378 strains were explainable with the results of the whole-genome identification, against aminoglycosides, β-lactams, fluoroquinolones, quinolones and tetracyclines. Only a limited number of antimicrobial agents, colistin and polymyxin B, remained as valuable options. However, the level of colistin resistance in *K. pneumoniae* is [33] rising rapidly worldwide. Therefore, additional control measures to address the emergence and spread of colistin-resistant CPKP must be taken seriously for the sake of public health.

Novel base pair substitution sites of the efflux pump genes *oqxA* and *oqxB* were identified in this study. While *oqxA* was found without any amino acid substitutions compared to a previously accessed sequence, *oqxB* showed 12-point mutations, resulting in a single substitution in the amino acid sequence from *oqxB26*. The genetic structure around the *oqxAB* gene was comparable to that of the *K. pneumoniae* strains accessed in previous studies [34, 35], with 99.35% identity and 100% query coverage, as revealed by a BLAST search. The OqxAB efflux pump is known to contribute to resistance against quinolones, tigecycline, and nitrofurantoin [36]. The phenotypic results of these strains showed corresponding resistance against quinolones but without tigecycline resistance, and nitrofurantoin resistance was not tested [13]. The chromosome-based efflux membrane transporter OqxAB was suggested to originate from *K. pneumoniae,* with a higher prevalence *in Klebsiella* spp. and *Enterobacter* spp*.* [37]. In South Korea, OqxAB was detected and reported from *K. pneumoniae* blood culture isolates collected between 2005 and 2010, but it was not detected in *Escherichia coli* isolates [38]. The work of Chen et al. in 2015 warned that the OqxAB operon could be mobilized via transposition events on mobile genetic elements, leading to the overexpression of efflux pumps [39]. Therefore, possible future synergism of *oqxAB* genes with MGEs such as plasmids and transposons must be considered and closely monitored.

In addtion, multiple variations in AMR gene sites, such as *ompK36*, *ompK37*, *acrR*, *gyrA* and *parC*, were revealed in this analysis. The functional differences of these varied gene products, such as OmpK36, are difficult to evaluate independently. However, mutation of the outer membrane porin at one or more gene sites is known to increase carbapenem resistance [40, 41]. The porin protein OmpK37 was suggested to have a narrower pore channel than OmpK36 and OmpK35, resulting in decreased susceptibility to β-lactam antibiotics [42]. In *K. pneumoniae*, deletion or alteration of outer membrane proteins is known to contribute to carbapenem resistance, especially when associated with ESBL or AmpC enzyme production [43, 44]. AcrR is known to bind to the promoter region of *acrA/acrR* and function as a negative modulator of the *acrAB* transcription level, leading to a decrease in the overexpression of *acrAB* [45, 46]. In other words, exclusion of *acrR* led to the upregulation of *acrAB* transcription [46]. Mutation of the repressor *acrR* was reported to have an effect on overexpression of *acrA* and *acrB* [47, 48]. Three amino acid sequence mutation sites were discovered in this sequencing of the *acrRAB* gene, which could result in the modification of the repressive function of *acrR* and overexpression of *acrAB*. Furthermore, the novel mutation sites were identified in the DNA topoisomerase-encoding sequence, with four regions in *gyrA* and one region in *parC*, which could lead to increased resistance through mutated drug target sites of fluoroquinolone agents. Therefore, the identified resistance mechanisms coupled in the ST378 CPKP strains must be monitored not only in human isolates but also in the animal isolates.

The whole-genome phylogenetic tree was constructed to measure the phylogenetic distance of ST378 *K. pneumoniae* strains from previously described isolates. Among the datasets, KPNIH48 (GenBank no. CP026392.1) was found to be phylogenetically close to the ST378 strains, which were isolated from the USA and typed as ST252. Similar resistance gene prevalence patterns were observable among closely related strains. However, the prevalence patterns of resistance genes and plasmid types were different for ST378 compared to neighboring strains. The ST378 *K. pneumoniae* has never been reported as a CPKP before. The identification of its genetic structure and gene distribution comparison using a whole-genome approach has revealed speculative coupling of multiple mechanisms contributing to resistance against various classes of antimicrobial agents. The epidemiologic analysis using a whole-genome approach in this study reveals the emergence of a novel strain of CPKP.

It is noteworthy that both ST378 strains have four heterogeneous plasmids (Fig. 4), and each plasmid harbors different resistance genes, instead of a single plasmid carrying multiple AMR genes. In the ST378 *K. pneumoniae*, the chromosome and three plasmids (Fig. 4A-C) were each identified to carry their own ESBL genes. The other plasmid was found to carry a carbapenemase gene. The characteristics of the plasmids carried by ST378 strains may indicate the process through which the ST378 strains have acquired MDR genes. Additionally, as reported in other *K. pneumoniae* strains, including the strains in this study, ST378 strains are expected to acquire further MGEs suitable for their survival.

In this study, two identified strains were analyzed due to the limited number of discovered isolates. The CPE surveillance system is not designed to adequately consider companion animals, even though they are closely associated with human society. Therefore, even if our study is not enough to represent our society, the genomic findings from these isolates should be considered seriously, because the subjects of this study are the first identified CPKP from companion animals in Korea. Both strains were identified from urine samples of dogs in Korea, which were suffering from urinary tract infections. The emergence of a novel CPKP strain, carrying various AMR genes coupled with MGEs, from a companion dog shows that CPKP poses a threat not limited to human health in our society. Considering the serious findings in this study, the unauthorized usage of carbapenems in veterinary clinics should be regarded as a serious concern.

## Conclusion

The whole-genome analysis of this study reveals the emergence of a novel strain of CPKP. The CPKP isolates in this study are characterized by their high resistance levels against various antimicrobial agents and their carriage of various AMR genes. Considering that the isolates are identified from companion animals, future control measures should be taken seriously from a "One Health approach" perspective.

### Supplementary Information

**Additional file 1: Table S1. **The whole genome profiles of sequenced *K. pneumoniae* isolates. **Table S2. **Previously tested MIC profiles of the DMCPKP1 and DMCPKP4. **Table S2. **Previously tested MIC profiles of the DMCPKP1 and DMCPKP4. **Table S4. **Gene identities of the antimicrobial resistance genes discovered from the whole-genome of 69 *K. pneumoniae* datasets. **Table S4. **Gene identities of the antimicrobial resistance genes discovered from the whole-genome of 69 *K. pneumoniae* datasets. **Table S6. **The pairwise SNP difference matrix of whole chromosome datasets extracted from the reference genome.

## Data Availability

All referred sequences of this study are available from the NCBI BioProject number PRJNA858561. All data generated or analyzed during this study have been submitted with this manuscript. All genetic information of the plasmids was deposited in GenBank. Therefore, all data from this study are available on public.

## Abbreviations

AMR: Antimicrobial resistance
CRE: Carbapenem-resistant Enterobacterales
CPE: Carbapenemase -producing Enterobacterales
CPKP: Carbapenemase-producing *K. pneumoniae*
ESBL: Extended-spectrum β-lactamase
iTOLs: Interactive tree of life
ML: Maximum likelihood
MIC: Minimum inhibitory concentration
MGEs: Mobile gene elements
MDR: Multidrug resistance
MLST: Multi-locus sequence typing
